# Effect of brief rest on hemodynamics and CSF oscillations across age

**DOI:** 10.1016/j.neuroimage.2025.121531

**Published:** 2025-10-15

**Authors:** Vidhya V Nair, Adam M Wright, Tianyin Xu, Elodie Foster, Xiaopeng Zhou, Yunjie Tong, Qiuting Wen

**Affiliations:** a Department of Radiology and Imaging Sciences, Indiana University School of Medicine, Indianapolis, IN, United States; b Weldon School of Biomedical Engineering, Purdue University, West Lafayette, IN, United States; c School of Health Sciences, Purdue University, West Lafayette, IN, United States

**Keywords:** Brief rest, Neurofluids, Low-frequency oscillations, Cardiac Pulsations, Fast fMRI

## Abstract

Brief eyes-closed rest (e.g., 30 min) during the day promotes rejuvenation and enhances mental clarity. However, its effect on neurofluid dynamics—including cerebral hemodynamics and cerebrospinal fluid (CSF) oscillations—remains largely unexplored. These neurofluid dynamics, driven by low-frequency oscillations (LFOs), respiration, and cardiac pulsation, play a critical role in the brain’s waste clearance and may serve as a key mechanism underlying the cognitive benefits of rest. This study used fast functional MRI (TR=363msec) to assess neurofluid dynamics across three frequency bands in five key regions of interest (ROI): cerebral arteries, superior sagittal sinus, grey matter, white matter, and fourth ventricle CSF. Measurements were taken before and after 30 min of eyes-closed rest in a cohort of 38 participants aged 35–82. After rest, we observed a significant increase in LFO power across all five ROIs, suggesting that enhanced LFO may promote neurofluid clearance and contribute to the rest’s restorative effects. Concurrently, cardiac power significantly decreased across all ROIs, indicating cerebrovascular relaxation, consistent with reduced cardiovascular activity during drowsiness. Lastly, older participants exhibited significantly smaller LFO and cardiac power changes than younger individuals, reflecting an age-related decline in neurofluid modulation that may diminish the benefits of rest. By simultaneously examining arterial, venous, and parenchymal hemodynamics, this study offers a integrated view of how brief rest influences brain pulsations and how these effects change with aging.

## 1. Introduction

Neurofluids,^1^ including cerebral blood and cerebrospinal fluid (CSF), play a key role in the brain’s waste clearance and have been shown to be modulated by changes in vigilance levels. For instance, both cerebral hemodynamics and ventricular CSF exhibit larger oscillation amplitudes during the initial light sleep period (Fultz et al., 2019; Helakari et al., 2022; Horovitz et al., 2008; Nair et al., 2025; Vijayakrishnan Nair et al., 2023). Recent animal studies further indicate that these increased oscillations facilitate waste clearance in the interstitial space (Holstein-Rønsbo et al., 2023; Jiang-Xie et al., 2024; Murdock et al., 2024). However, the effects of short-duration eyes-closed rest on neurofluid oscillations remain poorly understood. Brief resting sessions during the day are widely practiced to enhance mental clarity, improve cognitive performance, and reduce stress (Dutheil et al., 2021). These periods have been associated with spatial-specific decreases in alpha wave activity (Cantero et al., 2002) and a general shift in the autonomic nervous system toward parasympathetic dominance, fostering a calm state (Pressman and Fry, 1989). However, their effects on neurofluid dynamics—including cerebral hemodynamics and CSF oscillations—remain largely unexplored. Investigating these effects could provide new insights into the physiological mechanisms underlying the benefits of brief rest, particularly in relation to brain waste clearance.

Functional Magnetic Resonance Imaging (fMRI) is a powerful tool for investigating brain oscillations and has yielded important insights into brain dynamics during drowsiness. However, traditional fMRI acquisitions are limited by low temporal resolution, making it difficult to resolve faster physiological rhythms such as respiration and cardiac pulsations. As a result, most prior studies have focused primarily on low-frequency oscillations (LFOs: 0.01–0.1 Hz), reporting that drowsiness increases LFO power, signal variance (Chang et al., 2016; Fukunaga et al., 2006; Wong et al., 2016), and brain-wide functional connectivity (Korponay et al., 2024; Liu and Falahpour, 2020; Martin et al., 2021; Pourmotabbed et al., 2024).

Yet, neurofluid oscillations are not driven solely by LFOs. Emerging evidence highlights the crucial roles of respiratory (Birn et al., 2006; Nair et al., 2024) and cardiac pulsations (Greitz et al., 1992; Wright et al., 2024a) in modulating cerebrovascular and CSF dynamics (Vijayakrishnan Nair et al., 2022; Yang et al., 2022). All three of these oscillations have been shown to have a strong influence on neurofluids with distinctive dynamic spatial distributions over the brain during resting state wakefulness (Raitamaa et al., 2021). Drowsiness may affect each of these physiological rhythms differently, as it is associated with reductions in heart rate (Shinar et al., 2006) and ventilation (Colrain et al., 1987), potentially leading to frequency-specific shifts in neurofluid behavior that are not captured by LFO analysis alone. Recent advances in fast fMRI acquisition techniques now enable the simultaneous capture of LFO, respiratory, and cardiac frequencies. This expanded temporal bandwidth offers a unique opportunity to disentangle the contributions of distinct physiological drivers and to more accurately characterize neurofluid dynamics during drowsiness.

Neurofluid dynamics are not only temporally complex but also spatially compartmentalized by arteries, parenchyma hemodynamics, venous sinuses, and ventricles, each exhibiting unique oscillation patterns. For instance, larger cerebral arteries and dural sinuses primarily exhibit cardiac-driven pulsations (Hermes et al., 2023; Wright et al., 2024a, 2025), whereas parenchymal hemodynamics are dominated by low-frequency oscillations (Tong and Frederick, 2014). Changes in oscillation patterns within these larger arteries and sinuses—key components of upstream and downstream cerebral hemodynamic pathways—can offer valuable insights into the regulation of cerebral hemodynamics under varying vigilance levels. This is particularly relevant given their potential role in driving the perivascular CSF and facilitating brain clearance (Bojarskaite et al., 2023; Mestre et al., 2018; van Veluw et al., 2020). However, these distinct hemodynamic ROIs are often aggregated into a global fMRI signal, potentially masking region-specific effects during drowsiness. =Because LFO, respiration, and cardiac oscillations affect cerebral arteries and venous sinuses via different mechanisms, these regions may be differentially modulated by changes in vigilance. Therefore, mapping hemodynamic changes not only in parenchyma but also in its upstream and downstream blood circulations is essential for a better understanding of vigilance-related neurofluid dynamics.

The present study aimed to apply fast fMRI (TR = 363 msec) to investigate the effects of drowsiness—specifically, 30 min of eyes-closed rest—on brain oscillations across three different frequencies: LFOs, respiration, and cardiac pulsations. Using a recently developed data-driven approach for segmenting arteries and venous sinuses in fMRI (Wright et al., 2024b), we examined these oscillations across five ROIs: cerebral arteries, superior sagittal sinus (SSS), grey matter (GM), white matter (WM), and fourth ventricle CSF. The study included participants spanning a broad age range (35–82 years) to address two primary objectives: (1) to examine the common patterns of brain oscillation changes after 30 min of eyes-closed rest, and (2) to assess the influence of age on these changes.

## 2. Methods

### 2.1. Participants

Data from a total of 46 participants (35 female, 11 male) aged 35–82 (53.81±12.79) years were collected as part of this study, approved by Indiana University’s Institutional Review Board. Written informed consent was obtained from all participants before commencing the study procedures.

### 2.2. Data acquisition

**Fig. 1.**
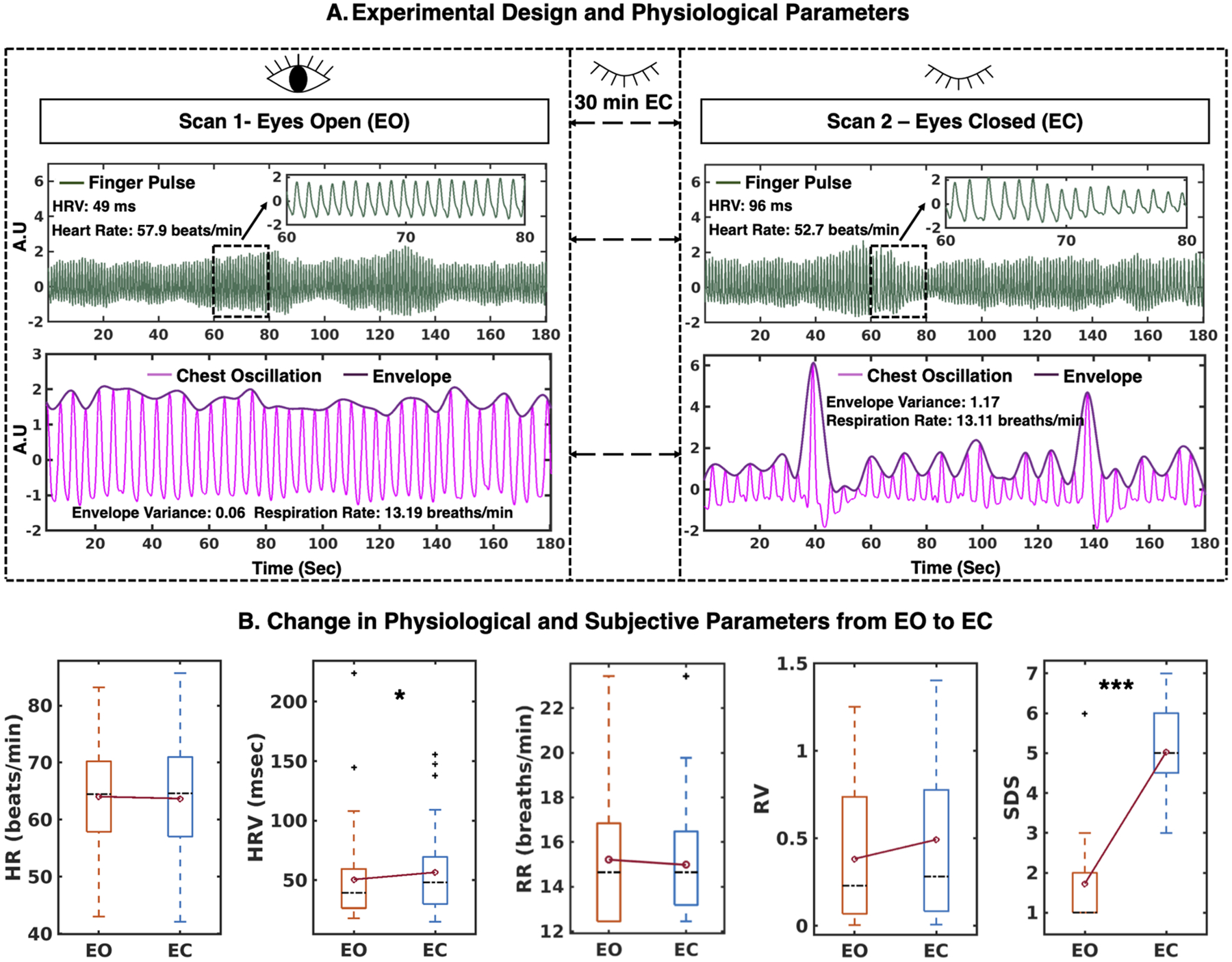
Overview of experimental design (A). Schematic of the experimental design, consisting of two fMRI scans separated by 30 min of uninterrupted eye closure. Physiological recordings from a representative participant are shown, including heart rate (HR) and heart rate variability (HRV) from finger pulse, as well as respiration rate (RR) and respiration variability (RV) from respiration recordings. (B). Changes in physiological parameters and subjective drowsiness score (SDS) between eyes-open and eyes-closed. *-*p* < 0.05; ***-*p* < 0.001; A.U-Arbitrary units; EO-Eyes-open; EC-Eyes-closed.

All imaging data were acquired using a 3T SIEMENS Prisma scanner with a 64-channel head-neck coil. The scans included structural T1-weighted MPRAGE (EPI with 2D MR acquisition, TR/TE: 2300/2.98 ms, flip angle: 9°, resolution: 1.0 mm × 1.0 mm × 1.0 mm) and two fMRI scans (TR/TE: 363/30 ms, flip angle: 35°, resolution: 2.5 × 2.5 × 2.5 mm^3^, number of volumes: 500, scan time: 3:02 min), one with participant’s eyes-open (EO) and one with eyes-closed (EC). A 30-minute interval of uninterrupted eye closure was incorporated between the two fMRI scans while the scanner completed other sequences (Fig. 1A). Physiological data, including respiration and finger pulse, were simultaneously recorded during the fMRI scans using the Siemens built-in physiological monitoring system. Extracted physiological metrics included heart rate (HR), heart rate variability (HRV, standard deviation of the R-R interval), respiration rate (RR), and respiration variability (RV, variance of the respiration envelope) (Fig. 1A). Following scan completion, participants were asked to rate their drowsiness level for both scans on an 8-point following the Stanford Sleepiness Scale (Shahid Azmeh and Wilkinson, 2012), where 1 corresponds to fully alert and 8 is fully asleep. After 30 min of rest, participants showed significantly higher HRV (paired Wilcoxon signed rank test, *p* = *0.04*) and subjective sleepiness scores (paired Wilcoxon signed rank test, *p* < *0.001*). Heart rate and respiration rate trended lower, while respiration variability trended higher, but these changes were not statistically significant (Fig. 1B).

### 2.3. Data preprocessing and quality check

All data pre-processing was performed using FSL (FMRIB Expert Analysis Tool, v6.07; Oxford University, UK (Jenkinson et al., 2012)) and MATLAB (The MathWorks Inc, 2023). The first 10 fMRI volumes were excluded to allow the signal to reach a steady state. Motion correction was intentionally omitted, as it can distort the slice-wise timing required for cerebral blood vessel segmentation and interfere with CSF inflow analysis. Specifically, the vessel segmentation method used in this study depends on matching the slice-wise fMRI signal timing to finger pulse plethysmography for retrospective cardiac alignment. Applying motion correction prior to this step would introduce interpolation across slices acquired at different time points, potentially distorting the temporal relationship between the fMRI signal and cardiac waveform. This issue is particularly problematic in interleaved acquisitions, where adjacent slices have substantially different acquisition times, leading to misalignment in the reconstructed time series (Parker and Razlighi, 2019). As motion correction was not applied in order to preserve slice timing integrity, we implemented rigorous motion artifact control procedures. Specifically, the FSL ‘*mcflirt*’ algorithm was used to calculate the absolute and relative displacements across fMRI volumes. fMRI data were excluded from further analysis if over 15 % of volumes had an absolute displacement greater than 1 mm or a relative displacement greater than 0.25 mm. Based on these criteria, data from 6 participants were removed due to significant motion. Data from an additional 2 participants were excluded due to the poor physiological recording quality. Consequently, the final analysis included data from 38 participants (27 females, 11 males; age range: 35–82 years, mean age: 53.18±13.49 years).

### 2.4. Generation of five fluid/tissue ROIs

**Fig. 2.**
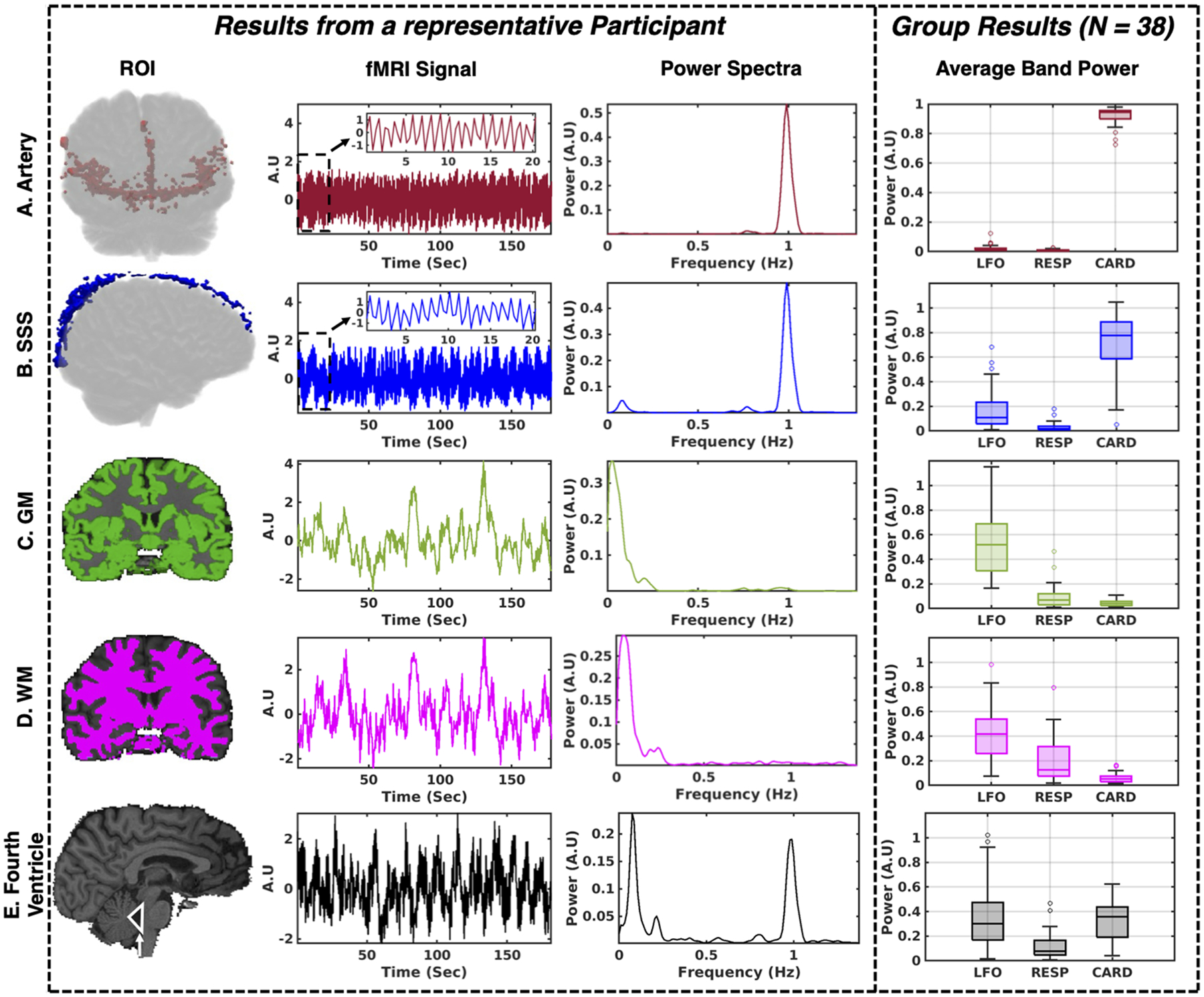
Illustration of five neurofluid ROIs with corresponding fMRI signals and their power spectra. Inset plots in **(A)** and **(B)** illustrate the cardiac pulsations in the artery and SSS, respectively. Boxplot of powers for three pulsations across all participants (*N* = 38), including LFO (0.01–0.1 Hz), Respiration (peak respiration frequency±0.05 Hz), and Cardiac pulsation (0.7–1.37 Hz). Abbreviations: A.U-Arbitrary units; SSS-Superior Sagittal Sinus; GM-Grey Matter; WM-White Matter; LFO-Low Frequency Oscillations.

Major cerebral arteries, including the middle cerebral artery, posterior cerebral artery, anterior cerebral artery, and the superior sagittal sinus (SSS), were segmented using a data-driven approach in the fMRI space (Fig. 2A, B) (Wright et al., 2024b). GM and WM masks were generated in the T1 space using ‘*fsl_anat’* (Fig. 2C, D) and subsequently registered to the fMRI space using FSL *‘flirt*’. The mean fMRI signal from each ROI was then extracted using ‘*fslmeants*’. CSF inflow signal was extracted from a single voxel in the middle of the fourth ventricle, identified on the T1-weighted image as previously described (Fig. 2E) (Fultz et al., 2019). All fMRI signals were detrended and demeaned before further analysis.

### 2.5. Power analysis of three pulsations

Power spectra were calculated for the mean fMRI signals in each ROI (MATLAB ‘*pwelch*’). Band power was then computed for three frequency bands: LFOs (0.01–0.1 Hz), respiratory (peak respiration frequency ±0.05 Hz), and cardiac (0.7–1.37 Hz) using MATLAB ‘*bandpower*’ function. This analysis was performed separately for eyes-open and eyes-closed scans to assess the effects of drowsiness.

### 2.6. Comparing changes in subgroups

To further investigate drowsiness-related effects, we categorized participants into “less drowsy” and “more drowsy” subgroups based on changes in physiology, anticipating larger brain pulsation changes in the latter. This subgroup analysis serves as a supplementary validation of findings observed across all participants. Specifically, participants were classified using a data-driven k-means clustering approach based on physiological and subjective drowsiness changes between eyes-open and eyes-closed scans. The clustering was informed by changes in five key measures: heart rate (ΔHR), heart rate variability (ΔHRV, standard deviation of R-R intervals), respiration rate (ΔRR), respiration variability (ΔRV, variance of the amplitude envelope of respiration), and subjective drowsiness score (ΔSDS) (see Note S1 in the supplemental material).

### 2.7. Statistical analysis

Statistical significance between conditions was tested using non-parametric Wilcoxon signed-rank tests. Multiple comparisons were corrected using the false discovery rate (FDR). Non-parametric effect size (Cliff’s δ) was also calculated. To investigate the effects of age and sex on drowsiness-induced power changes, linear regression was performed, modeling fMRI signal power change as a function of age and sex, using RStudio version 4.3.1. An additive model (i.e., fMRI signal power change ~ age + sex) was used, assuming that the main effect of age and the main effect of sex on the fMRI signal power change are separate, since the interaction between age and sex was found to be insignificant (see Note S2 in the supplemental material). The partial R^2^ was determined using the R function ‘*rsq.partial*’ within the rsq package.

## 3. Results

### 3.1. Spectral power distributions vary across ROIs

At resting state with eyes-open (Fig. 2-right panel), LFO power was highest in GM (0.52±0.25) and WM (0.43±0.23), moderate in fourth ventricle CSF (0.35±0.24) and SSS (0.17±0.17), and lowest in arteries (mean power=0.02±0.03). In comparison, cardiac power was dominant in arteries (0.92±0.06) and SSS (0.71±0.23), modest in fourth ventricle CSF (0.32±0.16), and least in WM (0.06±0.04) and GM (0.05±0.03). Respiratory power remained intermediate or low across all ROIs (WM: 0.19±0.18, Fourth Ventricle CSF: 0.11±0.10, GM: 0.09±0.09, SSS: 0.03 ±0.04, Artery: 0.009±0.006).

### 3.2. LFO power increases and cardiac power decreases after 30 min of eyes-closed rest

**Fig. 3.**
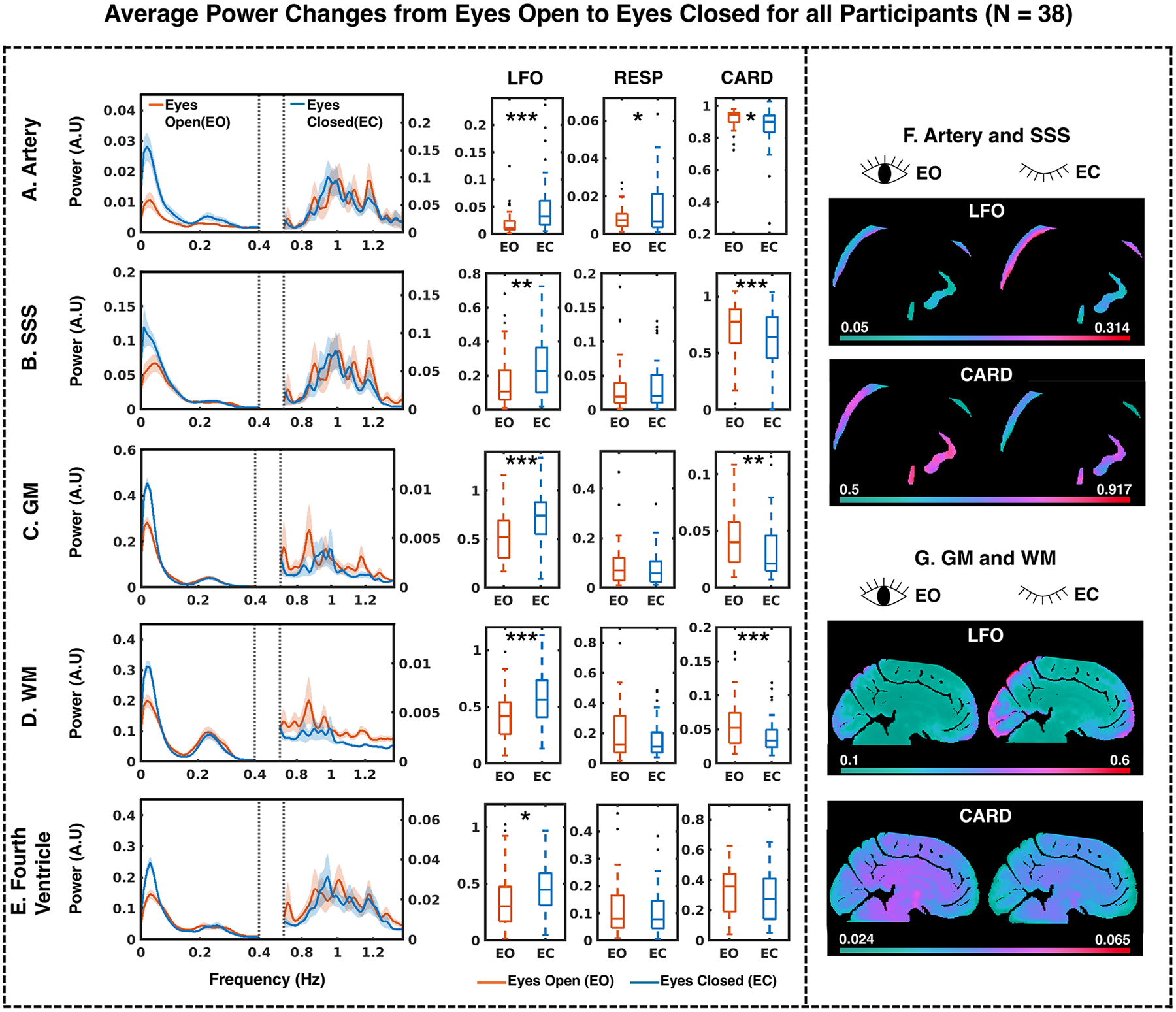
LFO power increases and cardiac power decreases after 30 min of eyes-closed rest across five ROIs. (A-E): Average power spectral changes before and after 30 min of eyes-closed rest for all participants in five ROIs. In each power spectrum, the thick line represents the mean, and the shaded region illustrates the standard error. (F-G): Voxel-wise power maps for LFO and cardiac (CARD) frequency bands, averaged across all participants. Abbreviations: *-*p* < 0.05; **-*p* < 0.01; ***-*p* < 0.001; A.U-Arbitrary units; SSS-Superior Sagittal Sinus; GM-Grey Matter; WM-White Matter; EO-Eyes-open; EC-Eyes-closed.

Fig. 3 shows changes in neurofluid oscillation power across all ROIs and frequencies (LFO, respiratory, cardiac) after 30 min of eyes-closed rest. The LFO power significantly increased in all regions after FDR corrections (GM: *p* < *0.001*, WM: *p* < *0.001*, Fourth ventricle CSF: *p* = *0.02*), SSS: *p* < *0.001*, Artery: *p* < *0.001*). In contrast, the cardiac power significantly decreased in all ROIs except the fourth ventricle (Artery*: p* = *0.02*, SSS: *p* < *0.001*, WM: *p* < *0.001*, GM: *p* = 0.001), fourth ventricle: *p* = *0.33*). Among all changes, the largest effect size was observed for the LFO power increase in arteries (Cliff’s δ: 0.46) and GM (Cliff’s δ: 0.43). Respiratory power did not exhibit consistent changes across all locations, with only a significant increase in arteries (*p* = *0.04*).

### 3.3. Power changes are more pronounced among the “More drowsy” participants

**Fig. 4.**
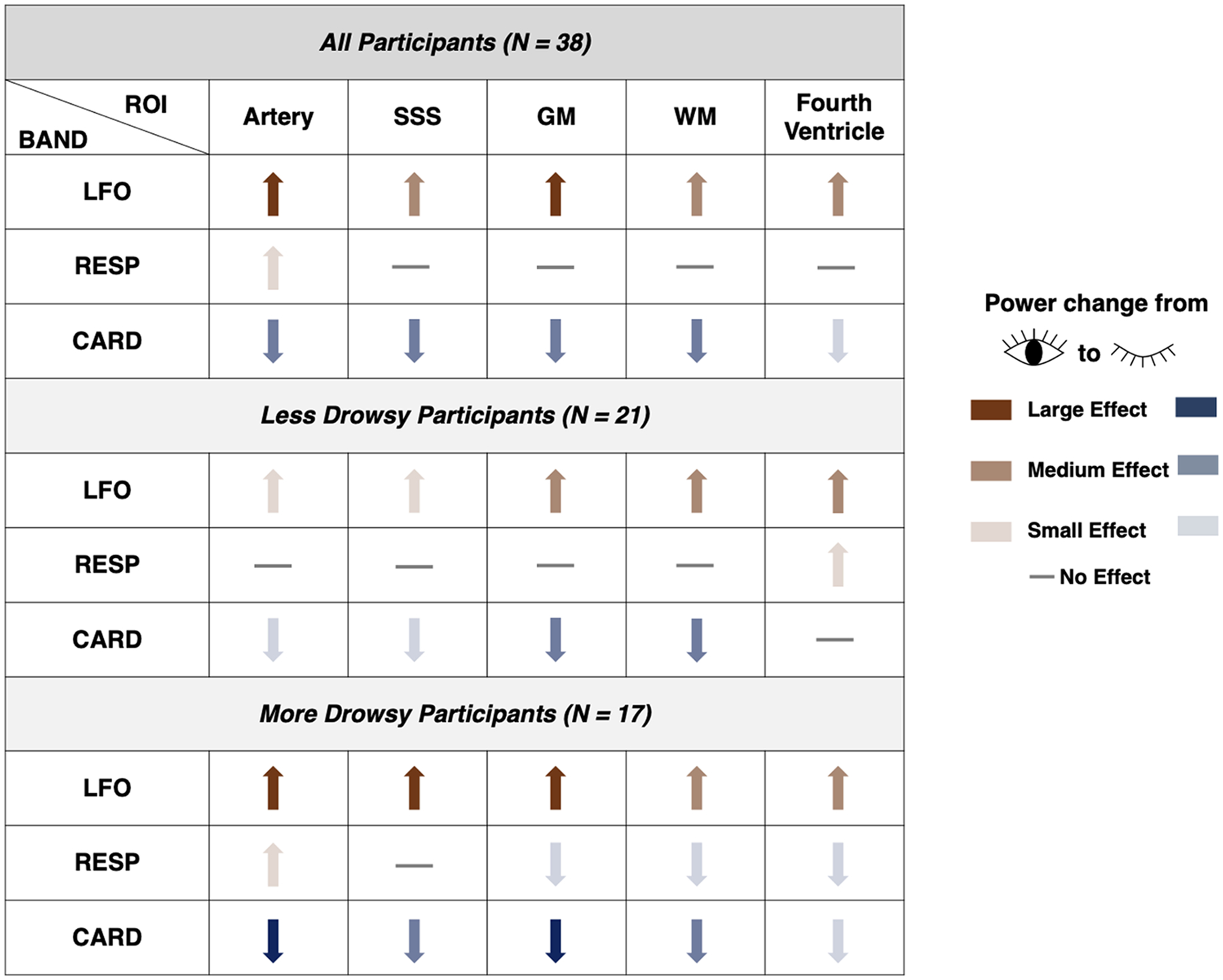
Summary of the effect size of power change after 30 min of eyes-closed rest among all participants (*N* = 38), less drowsy participants (*N* = 21), and the more drowsy participants (*N* = 17). Abbreviations: SSS-Superior Sagittal Sinus; GM-Grey Matter; WM-White Matter. Large effect size-Cliff’s δ≥0.43; Medium effect size-Cliff’s δ≥0.28; Small effect size-Cliff’s δ≥0.11; No effect-Cliff’s δ<0.11.

Fig. 4 summarizes the power changes after 30 min of eyes-closed rest across all participants (*N* = 38) and within two drowsiness-based subgroups: the less drowsy group (*N* = 21) and the more drowsy group (*N* = 17). The direction and effect sizes of these changes were illustrated with colored arrows to indicate large (Cliff’s δ≥0.43), medium (Cliff’s δ≥0.28), and small (Cliff’s δ≥0.11) effect sizes (Vargha and Delaney, 2000). Comparing the two groups, the increase in LFO power and the decrease in cardiac power were more pronounced among the more drowsy participants, with consistently larger effect sizes across all vascular ROIs. In contrast, respiratory power changes showed either a low effect size or no significant effect (Cliff’s δ<0.11) in both groups. These results confirm that the observed signal power changes are driven by drowsiness. A detailed description of power changes in the two subgroups is provided in Supplemental Note S3.

### 3.4. Aged brain exhibits smaller power changes

**Fig. 5.**
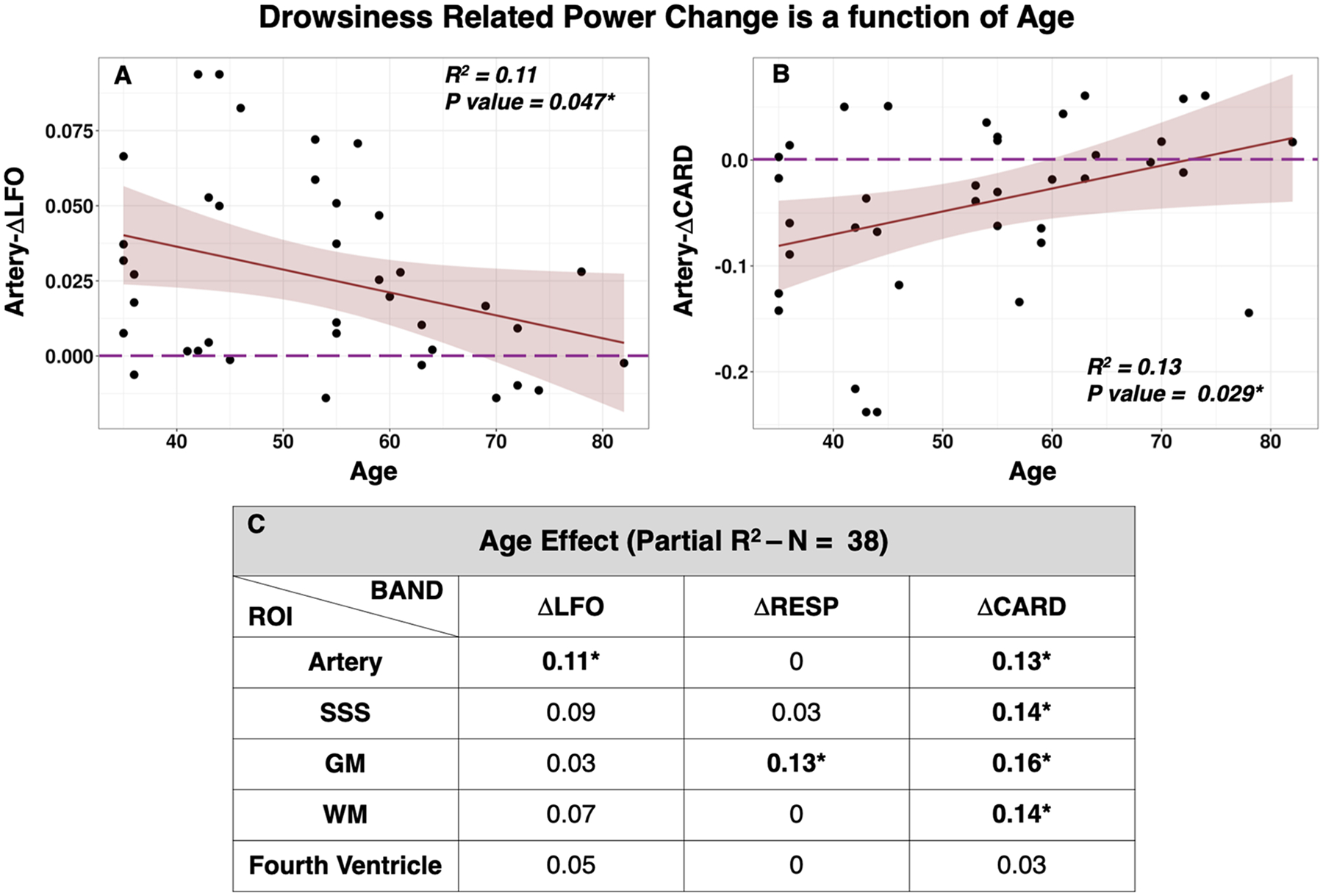
Aging is associated with smaller brain oscillation changes after 30 min of eyes-closed rest **(A-B):** Scatter plots illustrating the relationship between age and changes in arterial LFO (Artery-ΔLFO) and cardiac power (Artery-ΔCARD). The dark red line represents the regression curve, with the shaded red area representing the 95 % confidence interval. **(C):** Table showing the partial R^2^ for age for each multiple regression model. Abbreviations: ΔLFO/ΔRESP/ΔCARD - Change in LFO/respiration/cardiac power after 30 min of eyes-closed rest; *-*p* < 0.05; **-*p* < 0.01; SSS-Superior Sagittal Sinus; GM-Grey Matter; WM-White Matter.

Linear regression analysis revealed that older participants exhibit smaller power changes after 30 min of rest compared to younger individuals (Fig. 5). Specifically, arterial LFO power changes were more pronounced in younger participants but decreased with increasing age and approached the zero line (Fig. 5A, *p* = 0.047, R^2^=0.11). Similarly, cardiac power changes were larger in younger individuals but decreased in magnitude with age, a trend observed consistently across all vascular ROIs. These results suggest that older brains may be less responsive to drowsiness-related changes in physiological oscillation strength compared to younger brains. Sex was also found to have a significant effect on respiratory power change in the GM and WM. However, it might not be relevant as a drowsiness effect since the respiratory power change itself was insignificant after 30 min of eyes-closed rest (See Table S4 in the supplemental material).

## 4. Discussion

This study investigates the effects of 30 min of eyes-closed rest on neurofluid dynamics using fast fMRI (TR = 363 msec), enabling concurrent sampling of fluid oscillations across three frequencies in five ROIs. To our knowledge, this is the first study to assess these changes in key neurovascular ROIs, including cerebral arteries and the superior sagittal sinus. Our findings are twofold: First, eyes-closed rest led to a significant increase in LFO power and a concurrent decrease in cardiac power across all regions; Second, these changes were significantly attenuated in older participants. The physiological implications of these results are discussed below.

### 4.1. Increased LFO power after 30 min of rest

The increase in LFO power found in the current study aligns with reports of elevated fMRI global brain signal variance, LFO power, and global brain connectivity during periods of reduced vigilance (Chang et al., 2016; Fukunaga et al., 2006; Helakari et al., 2023; Korponay et al., 2024; Wong et al., 2016). Several mechanisms may contribute to this observation. First, shifts in neural activity during low-vigilance states may promote LFO oscillations. The transition from wakefulness to drowsiness is characterized by distinct EEG patterns, including increased theta power (4–7 Hz) and decreased occipital alpha power (8–13 Hz). These cortical EEG changes are strongly correlated with increased global fMRI signal fluctuations, suggesting a significant neural contribution to fMRI changes (Wong et al., 2016, 2013). Our findings extend this understanding by showing that LFO power increases not only in parenchymal tissue but also in arterial, venous, and ventricular regions. Second, autonomic nervous system modulation may further enhance cerebral LFOs. As vigilance declines, parasympathetic activity (associated with calm and rest) increases while sympathetic tone (associated with the fight-or-flight response) diminishes. This shift can amplify cerebrovascular LFOs through two pathways: direct vasodilation of cerebral vessels or indirect effects via systemic cardiovascular rhythms. Increased parasympathetic activity may directly induce cerebrovascular dilation via parasympathetic fiber innervation, thereby enhancing low-frequency oscillations (Boysen et al., 2009; Branston, 1995; Tamayo and Siepmann, 2021). Additionally, systemic effects of parasympathetic activity, such as increased heart rate variability—as observed in our participants—may promote LFOs throughout the vascular network, affecting both systemic and cerebral hemodynamics (Tong et al., 2019). Notably, these mechanisms likely interact, collectively shaping the observed increase in LFO power during drowsiness.

### 4.2. Decreased cardiac power after 30 min of rest

Interestingly, our results reveal that eyes-closed rest not only increased the LFO power but also resulted in a concomitant reduction in cardiac oscillation power across the various neurofluid ROIs. To our knowledge, this is the first study to report this observation. The reduced cerebral cardiac pulsation aligns with the diminished pulsatility in the central circulation during rest, driven by elevated parasympathetic activity. An increase in parasympathetic tone has been shown to induce a series of cardiovascular changes, including reduced heart rate, vascular dilations, and decreased both myocardial contractility (Freeman and Berger, 2024; Gordan et al., 2015) and cerebral artery contractility (Roloff et al., 2016). Taken together, this autonomic shift may attenuate cardiac-driven pulsations in the cerebral vasculature, promoting vascular relaxation during rest.

### 4.3. The aged brain shows smaller changes after 30 min of rest

Our findings reveal that, for the same duration of rest, older participants showed smaller changes in both LFOs and cardiac pulsations compared to younger individuals. This may be attributed to age-related alterations in autonomic nervous system function, slow-wave activity, and cerebrovascular integrity. With aging, sympathetic activity tends to increase while parasympathetic activity declines at rest, leading to a weaker sympathetic-to-parasympathetic shift during a 30min rest period compared to younger adults (Pfeifer et al., 1983). This attenuated autonomic adjustment likely contributes to the reduced vascular pulsation changes observed in older individuals. Moreover, age-related vascular changes may impair the ability of the cerebral vasculature to respond to neural and systemic mechanisms associated with drowsiness. Aging is associated with cerebral vessel stiffening, loss of vascular smooth muscle cells (Santisteban and Iadecola, 2025), and a reduction in capillary pericytes (Berthiaume et al., 2022). These structural and functional alterations likely contribute to the diminished brain oscillatory changes observed in older individuals. Taken together, both neuronal and vascular mechanisms may underlie the reduced modulation of brain oscillations during rest in the older brain.

### 4.4. Implications for the glymphatic system

The concurrent rise in LFO power and the decline of cardiac power across hemodynamic and CSF regions may have significant implications for glymphatic waste clearance. Short-duration eyes-closed rest is known to restore mental clarity and cognitive performance (Chen et al., 2020; Zare et al., 2023), and our results suggest a role for cerebrovascular LFOs in these effects. Among the three brain oscillation frequencies, LFOs have been particularly implicated in driving waste clearance in the interatrial space (Holstein-Rønsbo et al., 2023; van Veluw et al., 2020), with studies showing elevated LFOs during natural sleep in both animals (Bojarskaite et al., 2023; Hauglund et al., 2024) and humans (Fultz et al., 2019; Helakari et al., 2022; Vijayakrishnan Nair et al., 2023). Our study demonstrates that LFO elevation can occur brain-wide within just 30 min of eyes-closed rest, suggesting that even brief rest may promote glymphatic clearance and contribute to brain energy restoration. The concurrent reduction in cardiac pulsation power suggests that cardiac pulsation may play a relatively minor role in driving fluid clearance during rest, allowing the cardiovascular system to reduce high-frequency pumping and conserve energy. Lastly, our results indicate that older adults exhibit less pronounced changes in both LFO and cardiac pulsation in response to the same duration of rest, suggesting a diminished capacity for driving fluid clearance. This aligns with animal studies showing reduced glymphatic clearance during sleep in the aging brain (Benveniste et al., 2018; Kress et al., 2014; Voumvourakis et al., 2023). Overall, our findings demonstrate that brief rest enhances LFOs, which are critical to brain waste clearance. Additionally, this effect diminishes with age, reducing the strength of rest-induced oscillations, which may impact waste clearance function.

### 4.5. Limitations

Resting in the MR scanner may be inherently challenging due to the noisy environment. Therefore, the effects of short-duration rest on neurofluid dynamics may be more pronounced in a more comfortable setting than reported here. Additionally, our study did not include concurrent EEG recordings, which could have helped refine the analysis. For instance, EEG data could allow for better categorization of participants into more drowsy, less drowsy, and fully asleep groups or enable the examination of the direct relationship between EEG-derived drowsiness and fMRI power changes. This presents an opportunity for future improvement. Moreover, at a TR of 363 msec, the theoretical Nyquist frequency is 1.38 Hz. In participants with a higher heart rate and heart rate variability, the upper edge of the cardiac band may be undersampled, potentially leading to aliasing (Huotari et al., 2019). While we visually screened for such extreme cases, a higher sampling rate (shorter TR) would be preferable in future studies. Another limitation of this study is that motion correction was not applied in order to preserve the slice timing accuracy required for vessel segmentation. As a result, residual motion may influence signal dynamics, particularly in the LFO range. However, we consider this impact to be minimal given the rigorous motion exclusion criteria applied during data preprocessing.

### 4.6. Conclusions

In summary, the present study shows that 30 min of eyes-closed rest leads to a significant increase in LFO power alongside a notable decrease in cardiac power across hemodynamic and CSF regions. The increase in LFO power likely promotes fluid clearance, providing a potential explanation for why short-duration rest can help restore mental clarity. Conversely, the decrease in cardiac power suggests cerebrovascular relaxation during rest. Additionally, older participants exhibited significantly smaller changes in response to the same rest period compared to younger individuals, indicating a diminished restorative effect of rest in the aging brains.

## Supplementary Material

1

Supplementary material associated with this article can be found, in the online version, at doi:10.1016/j.neuroimage.2025.121531.

## Data Availability

All data and code used in this manuscript will be available upon reasonable request due to privacy/ethical restrictions.

## Footnotes

1 The term ‘neurofluids’ in this work refers to cerebral blood (Arteries, SSS, GM, and WM) and cerebrospinal fluid (Fourth Ventricle), though by definition, it also includes interstitial fluid (Agarwal et al., 2024).
