# Supplementary material for: Effect of brief rest on hemodynamics and CSF oscillations across age
Source: Neuroimage. Author manuscript; Available in PMC 2026 Jun 22. (PMC13285057; doi:10.1016/j.neuroimage.2025.121531)
Supplement: 1 [file NIHMS2184842-supplement-1.docx]

**Supplemental Material**

**S1. K-Means Clustering Analysis**

We relied on changes in physiological parameters (cardiac activity and respiration) as well as participant-reported subjective drowsiness scores (SDS) between eyes-open and eyes-closed scans to group participants into clusters based on their drowsiness levels – i.e., less drowsy and more drowsy. Accordingly, the change in physiological (HR, HRV, RR, and RV) as well as the change in SDS from eyes-open scan to eyes-closed scan, was calculated for each participant. Note that the change in these measures was determined by subtracting the eyes-open scan measure from the eyes-closed scan measure. The final features fed as inputs to the clustering algorithm (MATLAB *‘kmeans’*) include ΔHR, ΔHRV, ΔRR, ΔRV, and ΔSDS between eyes-open and EC scans (Figure S1A).

**Figure S1:** **(A).** A flowchart illustrating the approach for classifying participants into two clusters based on drowsiness level. **(B).** Importance is given to different input features by the K Means clustering algorithm. **(C).** Change in input features fed into the K Means algorithm between the two clusters and change in physiological/subjective measures from EO to EC separately in cluster 1 in **(D)** and cluster 2 in **(E)**. **^•^**-p approaching significance; ***-p<0.001; A.U-Arbitrary units; HR-Heart Rate; HRV-Heart Rate Variability; RR-Respiration Rate; RV-Respiration Variability; SDS-Subjective Drowsiness Scores; EO-Eyes-open; EC-Eyes-closed.

K Means algorithm, when supplied with change in physiology/subjective measures from eyes-open to eyes-closed conditions, grouped the participants into two clusters – less drowsy cluster 1 (N = 21) and more drowsy cluster 2 (N = 17). The importance given by the K Means clustering algorithm to the different input features can be found in Figure S1B. The algorithm assigns the highest importance to change in heart rate variability (ΔHRV, as is evident from the significantly larger ΔHRV in cluster 2 compared to cluster 1 (Figure S1C)), followed by ΔHR, ΔRV, ΔSDS, and ΔRR.

Comparison between the two clusters shows that when going from eyes-open to eyes-closed, cluster 2 has a larger average decrease in heart rate and respiratory rate as well as a larger average increase in heart rate variability, respiratory variability, and subjective drowsiness scores in relation to cluster 1 (Figure S1C), thereby revealing a higher level of drowsiness in cluster 2. Furthermore, it can also be seen that the direction of change in these indices from eyes-open to eyes-closed is more revealing of an increasing drowsiness level in cluster 2 compared to cluster 1 (Figure S1D and S1E).

**S2. Interaction Effects between Age and Sex on fMRI Signal Power Change from Eyes-open to Eyes-closed**

The final analysis presented in the study included data from 27 females (Age range: 35-82 years, Mean±SD: 53.93±13.36 years) and 11 males (Age range: 35-74 years, Mean±SD: 51.36±14.31 years). It can be seen that our data has been asymmetric sex distribution across the entire age range of 35-82 years and therefore may not be ideal to study the age-sex interaction effect. For this reason, the current results were estimated based on an additive model (i.e., fMRI signal power change ~ age + sex), assuming that the main effect of age and the main effect of sex on the power change are separate. To verify this, we also ran an interaction model (i.e., fMRI signal power change ~ age * sex), to see if the age-sex interaction becomes a significant effect on power change. The results of these analyses are presented below in Table S2. It can be seen that this age-sex interaction effect is significant only for the respiratory power change in SSS. However, since this change in respiration range power between eyes-open and eyes-closed in SSS itself is not significant, this interaction effect is not interpretable.


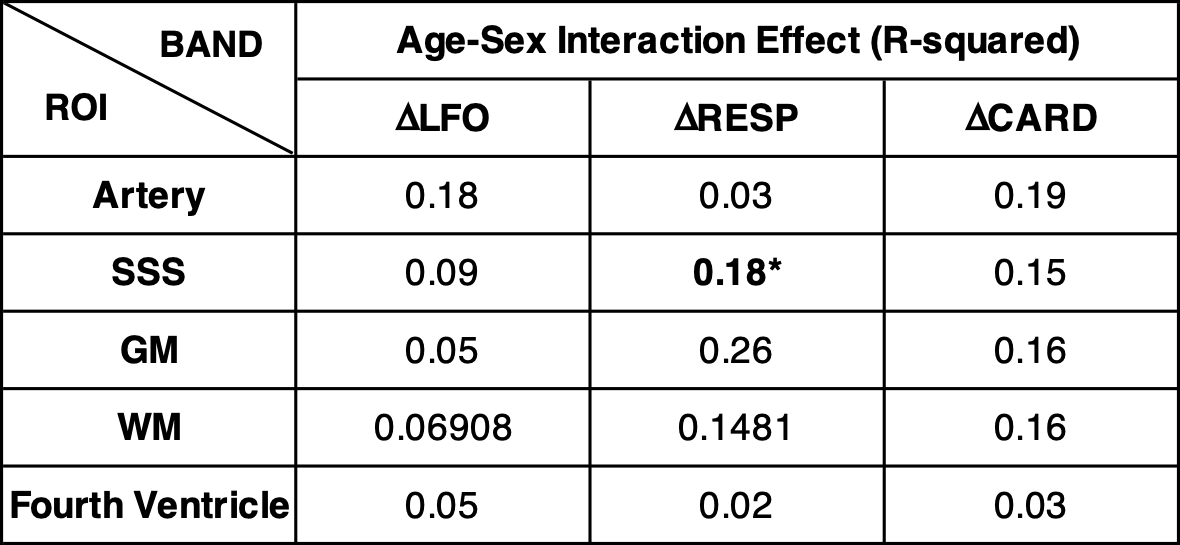


*Table S2: Table showing the R^2^ for age-sex interaction effect for each multiple regression model. Abbreviations: ΔLFO/ΔRESP/ΔCARD - Change in LFO/respiration/cardiac power after 30 minutes of eyes-closed rest; *-p<0.05; SSS-Superior Sagittal Sinus; GM-Grey Matter; WM-White Matter.*

**S3. Average Power Changes from Eyes-Open to Eyes-Closed between the Clusters**

Figure S2, shown below, illustrates the average power changes from eyes-open to eyes-closed between the two clusters. The less drowsy cluster exhibited a significant increase in LFO power from eyes-open to eyes-closed only in GM (*p<0.01*) and WM (*p<0.01*) while a significant decrease in cardiac power was observed in WM (*p<0.05*). On the other hand, participants in the drowsier cluster showed significant increases in LFO power as well as a significant decrease in cardiac power from eyes-open to eyes-closed across arteries (*p<0.01* for LFO increase and cardiac decrease), SSS (*p<0.05* for LFO increase and cardiac decrease), GM (*p<0.05* for LFO increase and *p<0.01* for cardiac decrease), and WM (*p<0.05* for LFO increase for cardiac decrease). A significant change in respiratory power was observed in GM (*p<0.05*) only for the drowsier cluster.


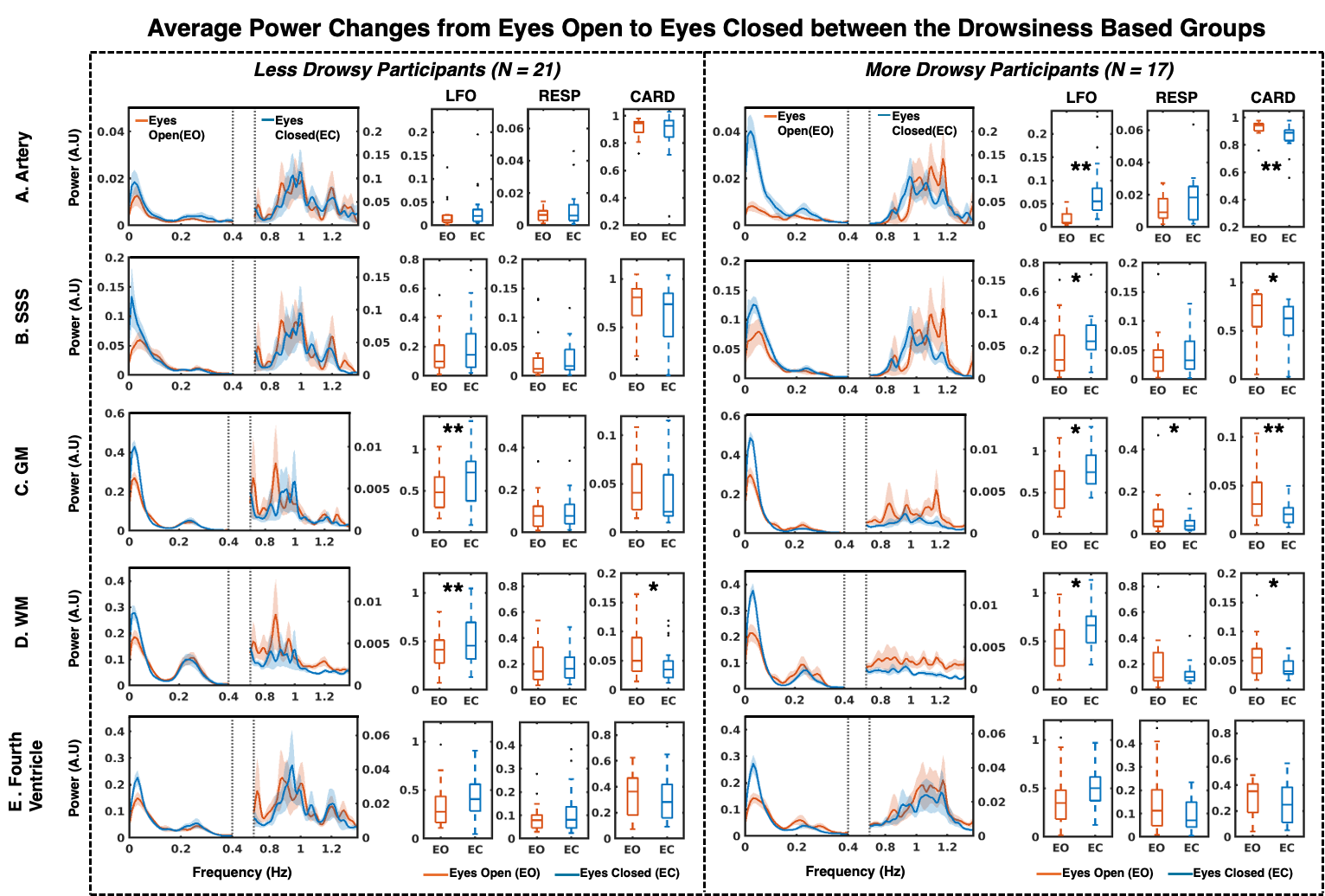


***Figure S3:*** *Average power spectral changes from EO to EC for less drowsy(left) and more drowsy(right) clusters. LFO power increase and cardiac power decrease in moving from EO to EC were more evident in the drowsier cluster across all regions of interest. In power spectral curves, the thick line represents the mean, and the shaded region illustrates the standard error. *-p<0.05; **-p<0.01; ***-p<0.001; A.U-Arbitrary units; SSS-Superior Sagittal Sinus; GM-Grey Matter; WM-White Matter; EO-Eyes-open; EC-Eyes-closed.*

**S4. Influence of Sex on fMRI Signal Power Change from Eyes-open to Eyes-closed**

**
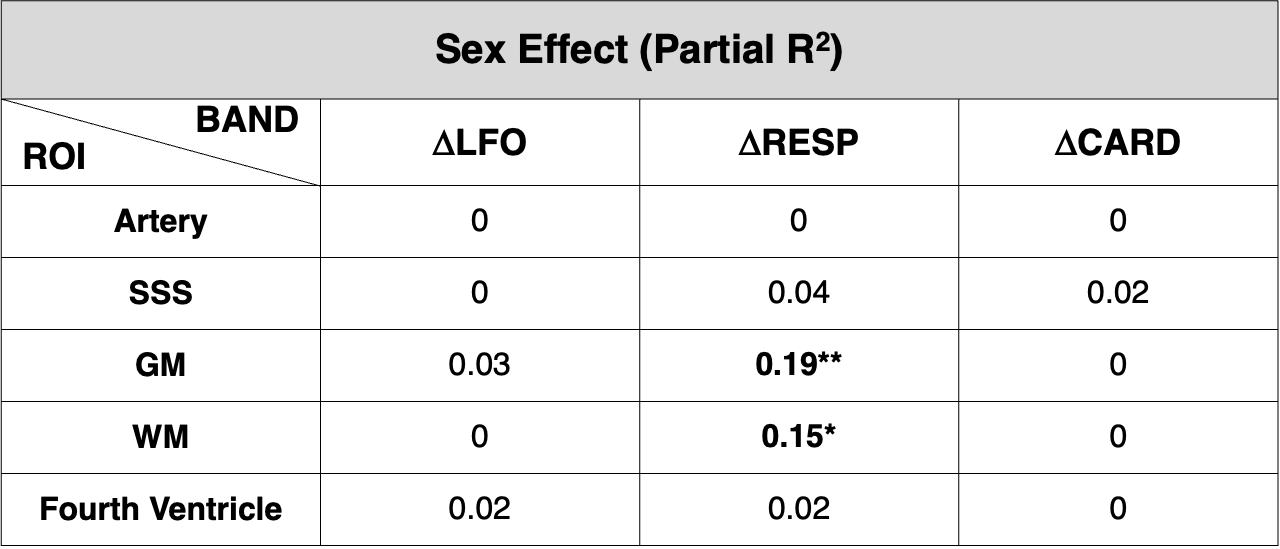
**

*Table S4: Table illustrating the partial R^2^ for sex for each multiple regression model. *-p<0.05; **-p<0.01; SSS-Superior Sagittal Sinus; GM-Grey Matter; WM-White Matter.*
